# Seasonal dynamics of the genus: *Planktoniell*a Schutt in the estuarine waters of Indian Sundarbans

**DOI:** 10.1186/s40064-016-2195-4

**Published:** 2016-05-12

**Authors:** Sanoyaz Sekh, Biswajit Biswas, Manjushree Mandal, Neera Sen Sarkar

**Affiliations:** Phycology Section, Department of Botany, University of Kalyani, Kalyani, Nadia, West Bengal 741235 India

## Abstract

The study highlights the dynamics and morphological characteristics of the Genus *Planktoniella* Schutt. The two available species *P. sol* (Wallich) Schutt. and *P. blanda* (Schmidt) Syvertsen and Hasle are important components of the phytoplankton assemblage in the estuarine system of Indian Sundarbans and also marine systems elsewhere. The sampling sites for the purpose of this study include four different spots along a riverine stretch in the estuarine region adjacent to the Tiger Reserve in the Indian Sundarbans flowing into the Bay of Bengal. Integrated phytoplankton samples were preserved for the purpose from composite water samples from each site. The water samples were analysed in field for determining pH, temperature, salinity, conductivity, TDS, turbidity and DO and subsequent to treatment and processing, the samples were microscopically analysed in the laboratory. Significant negative correlation of cell count of both species found with respect to temperature and turbidity. *P. sol* versus temperature (significant at α = 0.01, p = 0.001) and *P. blanda* versus temperature (significant at α = 0.05, p = 0.037); *P. sol* versus turbidity (at α = 0.05, p = 0.019) and *P. blanda* versus turbidity (at α = 0.05, p = 0.019). Significant positive correlation found with respect to DO and as correlation between the two species themselves. A model has been generated for each of the two species with temperature, turbidity and DO as predictor variables and the two species of *Planktoniella* as response variables. The influence of other dominant phytoplankton in the samples has also been considered with Pearson correlation computed for each set of species.

## Background

Mangroves and estuaries are ecosystems known to be extremely sensitive to environmental fluctuations and are under perpetual stress because of varied reasons, and are thus particularly vulnerable to climate change. Significant environmental factors that affect the structure and function of these systems are expected to be sensitive parameters of global climate change and contribute to our understanding of the implications that such changes may have in the area under study. The present study area—the Sundarbans, is an interesting ecosystem for studying such changes. *Planktoniell*a Schutt is essentially a warm water diatom reported from tropical waters of marine and estuarine systems (Hasle and Syvertsen 1997; Romero et al. 2002; Balkis 2008; Romero et al. 2009; Biswas et al. 2010; Manna et al. 2010; Mukherjee et al. 2013), with some sporadic records of *Planktoniella sol* from Atlantic waters in the Norwegian Sea along the Norwegian west coast (Hasle and Syvertsen 1997). Nevertheless, Round (1981) considered the species *P. sol* to be a true tropical element and also suggested that it may have greater temperature tolerance compared to most tropical species (Round 1973). The genus has generally not been reported as bloom forming algae and accounted relative abundance (%) varies between 0.07 ± 0.121 and 1.317 ± 1.929 in case of *P. sol* and between 0.989 ± 2.039 and 1.33 ± 1.066 in case of *Planktoniella blanda* in the Indian Sundarbans (Biswas et al. 2010), though report of *P. sol* being part of bloom forming event (Rajasekar et al. 2010) or as a dominant component in ‘milky sea’ samples (Lapota et al. 1988) are infrequently found. Yearlong presence of the species has not been reported, instead it appears being to be present seasonally (Manna et al. 2010). The genus finds mention in a number of publications as being part of the diet composition of fish (Indira et al. 2013; Priyadharsini et al. 2014), crab (Nakhodai et al. 2013) and zooplankton (Schnetzer and Steinberg 2002), emphasising its value in the aquatic trophic system.

The genus is characterised by the presence of organic extrusions from the girdle which are of different morphological types. This renders identification of the species difficult after the normal frustules cleaning procedures, which removes organic material during acid cleaning and mounting, leading to misidentification of the genus as *Thallassiosira* spp. (Hasle and Syvertsen 1997). Two species—*P. sol* and *P. blanda* find ephemeral mention in enumeration lists of different publications from the Indian Sundarbans (Manna et al. 2010; Biswas et al. 2010; Mukherjee et al. 2013). Although, in these records, neither species was described adequately to confirm their identification and presence in the estuarine system of Indian Sundarbans. The present work gives attention to the morphometry and taxonomy of the two species along with an analysis of the changing ecological dynamics in terms of seasonal data as well as an analysis of their temporal factsheets obtained as secondary data.

## Methods

### Study area

**Fig. 1:**
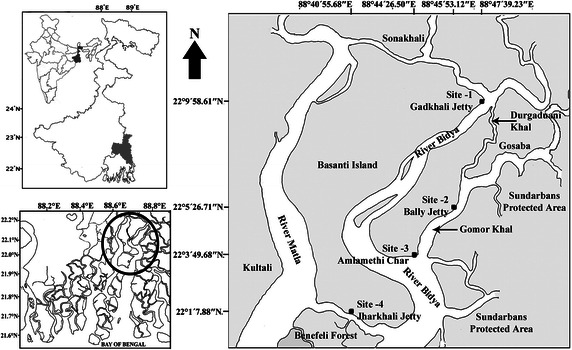
Sampling sites in the Indian Sundarbans

The Indian Sundarbans, which is geographically contiguous with a larger but ecologically-similar expanse in Bangladesh, is known to be the largest single deltaic tract of mangrove forests in the world. Four sampling sites have been part of this study on the estuarine stretch of the river Bidya which flows north to south adjacent to the Sundarban Tiger Reserve into the Bay of Bengal and is connected with another major river Matla through a small connecting river Herobhanga which flows in the east–west direction between Benefeli Forest of the 24 Parganas (South) Forest Division and Jharkhali (Fig. 1). This particular river stretch is of ecological interest since one bank is highly populated with obvious anthropogenic pressures and the opposite bank is that of the Reserve Forest of the Sundarban Tiger Reserve. Mid river collections were made for the purpose at the 4 different sampling spots along the river Bidya. The GPS coordinates of the sampling sites were noted using a hand held GPS (Garmin, Model: Oregon 550). The GPS coordinates of the sampling sites were: Site 1: Durgaduani river junction opposite to Gadkhali Jetty (22°9′58.61′′N and 88°47′39.23′′E); Site 2: Dattar/Gomor *khal* adjacent to Bally jetty (22°5′26.71′′N and 88°45′53.12′′E) ‘*khal’* is the vernacular for a small and narrow river.; Site-3: River Bidya adjacent to Amlamethi *char* (22°3′49.68′′N and 88°44′26.50′′E) ‘*char*’ is the vernacular for a newly silted up land-mass; and Site 4: Herobhanga river adjacent to Jharkhali jetty (22°1′7.88′′N and 88°40′55.68′′E).

### Sampling

Collections were made during March, 2013 to April, 2014 covering 4 dominant seasons of Sundarbans: summer (March–May), monsoon (June–August), post monsoon (September–November) and winter (December–February). Composite water samples (50 litres each) were collected from the collection boat at each of the sites and integrated phytoplankton samples were preserved with the addition of Lugol’s iodine followed by consecutive centrifugation and decantation to a fixed volume of 25 ml with 3 replicates at each site of collection during every season. Sampling for water parameter studies was also performed simultaneously.

### Analysis

Water parameter analysis was performed in the field using a portable Multi-Parameter Analyser Kit (Systronics, Model: 371). The biological analysis was performed in the laboratory. Subsequent to treatment and processing, the plankton samples were microscopically analysed in the laboratory using a Neoplan-N-TRF Fluorescence & Phase Contrast Microscope (Getner) with CCD Imaging System. Statistical analyses were performed using PAST version 3.02 and Minitab 17 software.

## Results and discussion

### Taxonomic treatment

The two species of *Planktoniella* under consideration have been analysed taxonomically following Guiry and Guiry (2015), Lee et al. (2012), Al-Kandari et al. (2009), Balkis (2008), Hasle and Syvertsen (1997), Pillai and Gopinathan (1973) and Durairatnam (1964). The classification system followed here is based on Round et al. (1990) and Medlin and Kaczmarska (2004) which is reflected in the Algaebase classification (Guiry and Guiry 2015)

Phylum: Bacillariophyta

Subphylum: Bacillariophytina

Class: Mediophyceae

Subclass: Thalassiosirophycidae

Order: Thalassiosirales

Family: Thalassiosiraceae

Genus: *Planktoniella* Schütt 1892

Type species: *Planktoniella sol* (Wallich) Schütt

Type designation: Schütt (1892): 258, Fig. 64

Centric diatoms with discoid cells. Areolae seen in radial or tangential rows. Organic extensions of the girdle are characteristic of this genus. Presence of a central strutted process (fultoportula) and one or two labiate processes (rimoportula) and a ring of marginal processes reported but not clearly visible under light microscope.

The identification of the genera poses problem since the organic extensions usually disappear after frustule cleaning treatment that diatoms are subjected to for microscopic observation. Under such circumstances the genera tends to get misidentified as *Thalassiosira* sp. (Hasle and Syvertsen 1997). As such observations need to be made before cleaning the frustules as simple water mounted slides. But though this marine genus has both fultoportulae and rimoportulae, and could have been included in *Thalassiosira*, it is preferable to maintain the two genera separately, on the basis of the extended wing since the genus is already large and extremely diverse.

#### Key to species found in the Indian Sundarbans

1a. Girdle with a continuous flap like membranous wing…………………. *P. sol* (Wallich) Schutt

1b. Girdle with mucilaginous lobes……………………….. *P. blanda* (Schmidt) Syvertsen & Hasle

**Plate 1:**
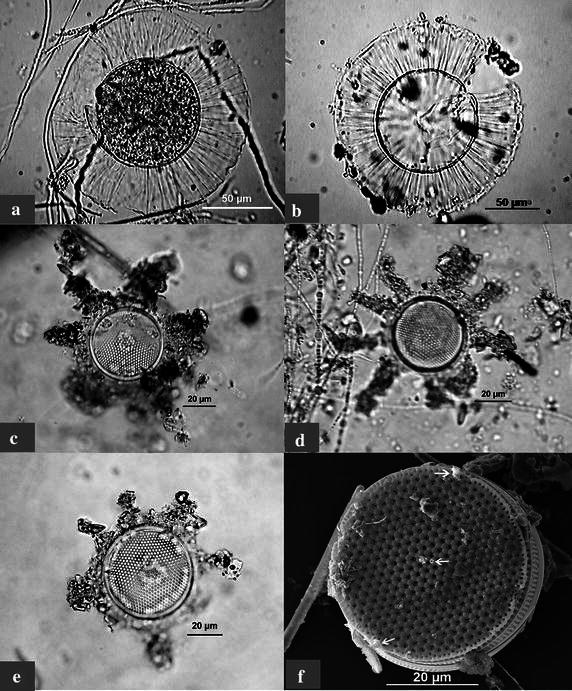
**a**, **b** *Planktoniella sol* (LM); **c**–**e** variations in organic lobe extensions of *Planktoniella blanda*; **c**, **e** *Planktoniella blanda* with 6 lobes (LM); **d** *Planktoniella blanda* with 9 lobes (LM); **f** *Planktoniella blanda* showing (*white arrows*) two marginal labiate processes—rimoportulae and a single central strutted process—fultoportula (SEM)

1a. *Planktoniella sol* (Wallich) Schutt **(Plate**1, **figs. a & b)**

Type designation: Schütt (1892): 258, fig. 64

Basionym: *Coscinodiscus sol* Wallich

References: Wallich (1860): 38, fig. 2(1); Schmidt (1878): fig. 59 (35–37); Schütt (1892) : 258, fig. 64; Desikachary (1989): 9, fig. 742–744; Hasle and Syvertsen (1997): 39–41, fig. 2.

Solitary cells, discoid, entire wing-like ribbed expansion of organic material extending from valve mantle. Central part characterised by convex to flat valves with polygonal areolation arranged as tangentially curved striae. Chromatophores dispersed within the valve face. Valve diameter of the central valve 70–95 µm, including the extended wings 130–175 µm.

**1b.***Planktoniella blanda* (A.Schmidt) E.E.Syvertsen & G. R. Hasle **(Plate** 1, **figs**. **c**, **d**, **e** & **f**)

Basionym: *Coscinodiscus blandus* A.Schmidt

Synonimised names: *Coscinodiscus blandus* Schmidt 1878

*Coscinodiscus bipartitus* Rattray 1890

*Coscinodiscus latimarginatus* Guo 1981

*Thalassiosira blanda* (Schmidt) Desikachary and Gowthaman, 1989

*Thalassiosira bipartita* (Rattray) Hallegraeff 1992

References: Schmidt (1878): fig. pl. 59 (35–37); Desikachary (1989): fig. pl. 742 (1–7), 743 (1–5), 744 (1–5); Hasle and Syvertsen (1997): 39–41, fig. 2.

Solitary cells, discoid, lobes of organic material extending from valve mantle. Lobes vary from 6 to 9 in number in the observed organisms. Valve face flat with tangential straight striae. A single central strutted process and two labiate marginal processes visible in SEM. Chromatophores dispersed within the valve face. Valve diameter of the central valve 34–50 µm, including the extended lobes 50–72 µm.

### Analysis of seasonal dynamics of physico-chemical parameters

All water chemistry data has been summarized in Table 1. Annual average range in water temperature is between 24.35 ± 6.43 °C (Dec.–Feb.) to 32.25 ± 1.48 °C (Mar.–May) at site 4. Maximum variations in pH values are also noted at site 4 during Mar.–May (7.42 ± 1.63) and Dec.–Feb. (8.90 ± 0.19). Annual variation in salinity averages gives highest value during Mar.–May at site 4 (20.40 ± 2.69 ppt) and lowest value during Sep.–Nov. at site 1 (11.82 ± 2.95 ppt), the highest standard deviation of 6.29 is noted during Jun.–Aug. at site 2. Among all the parameters, turbidity—a measure of suspended solids in the aquatic system, shows the highest range, varying between 25.00 ± 24.00 and 395.00 ± 177.00 NTU with lowest turbidity observed during Dec.–Feb. and highest during Jun.–Aug. at all four sites. Highest variation in TDS (Total Dissolved Solids) is observed at site 1 with maximum value during Mar.–May (19.95 ± 3.32 NTU) and lowest during Dec.–Feb. (15.25 ± 5.44 NTU). Conductivity as a parameter does not exhibit much variation on neither temporal nor spatial scale and maximum variation is noted during Mar.–May at site 1 varying between 20.35 ± 1.77 and 36.35 ± 6.67 mS/cm. Lowest DO values were recorded during Jun.–Aug. and Sep.–Nov. with the minimum being 5.75 ± 0.77 mg/l at site 3 during Jun.–Aug. with the maximum value of DO (8.70 ± 3.54 mg/l) also at site 3 during Dec.–Feb.

**Table 1:** Seasonal variation in mean ± standard deviation of water physico-chemical parameters from the four sampling sites

Season and site		Temperature (°C)	pH	Salinity (ppt)	Turbidity (NTU)	TDS (mg/l)	Conductivity (mS/cm)	DO (mg/l)
Mar.–May	1	31.80 ± 1.56	8.50 ± 0.69	18.35 ± 3.61	75.00 ± 49.50	19.95 ± 3.32	32.10 ± 14.4	6.07 ± 1.37
	2	31.25 ± 2.62	8.55 ± 0.32	14.30 ± 4.10	76.50 ± 30.40	17.65 ± 6.01	36.35 ± 11.7	6.52 ± 1.02
	3	31.45 ± 1.91	8.75 ± 0.25	20.35 ± 1.77	53.00 ± 32.50	19.80 ± 3.68	20.35 ± 1.77	6.33 ± 1.04
	4	32.25 ± 1.48	7.42 ± 1.63	20.40 ± 2.69	61.00 ± 29.70	18.75 ± 4.17	33.00 ± 13.01	6.80 ± 2.26
Jun.–Aug.	1	31.85 ± 0.91	7.71 ± 0.43	12.30 ± 1.56	395.0 ± 177.0	16.85 ± 2.76	26.00 ± 6.51	6.00 ± 1.13
	2	32.05 ± 1.20	7.84 ± 0.28	16.25 ± 6.29	355.0 ± 106.1	18.50 ± 3.96	33.9 ± 13.15	5.9 ± 0.707
	3	31.85 ± 1.48	7.79 ± 0.30	14.55 ± 5.16	380.0 ± 99.00	18.50 ± 3.82	30.90 ± 14.40	5.75 ± 0.77
	4	32.05 ± 1.06	8.19 ± 0.43	13.95 ± 3.32	335.0 ± 134.4	18.00 ± 3.96	26.10 ± 7.64	6.15 ± 1.20
Sep.–Nov.	1	31.75 ± 1.63	8.44 ± 0.47	11.82 ± 2.95	108.5 ± 101.1	15.45 ± 5.73	26.10 ± 3.96	5.85 ± 0.63
	2	30.35 ± 1.34	8.34 ± 0.12	12.30 ± 1.56	126.0 ± 48.10	15.60 ± 3.11	26.90 ± 2.40	6.05 ± 0.21
	3	31.85 ± 0.49	8.45 ± 0.32	12.85 ± 1.91	104.4 ± 78.50	16.05 ± 3.46	23.95 ± 2.33	6.10 ± 0.42
	4	30.35 ± 2.05	8.44 ± 0.45	13.25 ± 2.05	88.50 ± 30.40	16.45 ± 3.89	24.00 ± 2.97	6.50 ± 0.18
Dec.–Feb.	1	27.50 ± 3.54	8.26 ± 0.21	12.32 ± 3.66	25.00 ± 24.00	15.25 ± 5.44	27.15 ± 5.44	7.70 ± 3.25
	2	27.00 ± 2.83	8.72 ± 0.41	14.50 ± 4.38	80.50 ± 16.30	17.25 ± 5.16	30.60 ± 7.64	8.70 ± 3.54
	3	28.25 ± 5.59	8.89 ± 0.30	14.55 ± 4.31	28.60 ± 28.80	17.25 ± 5.16	30.80 ± 7.35	8.50 ± 3.25
	4	24.35 ± 6.43	8.90 ± 0.19	14.70 ± 4.10	37.50 ± 41.80	17.05 ± 4.74	30.65 ± 6.58	8.05 ± 2.33

Site 1: Gadkhali (Durgaduani khal); Site 2: Bally (Gomor khal); Site 3: Amlamethi (River Bidya); Site 4: Jharkhali (River Herobhanga)

**Fig. 2:**
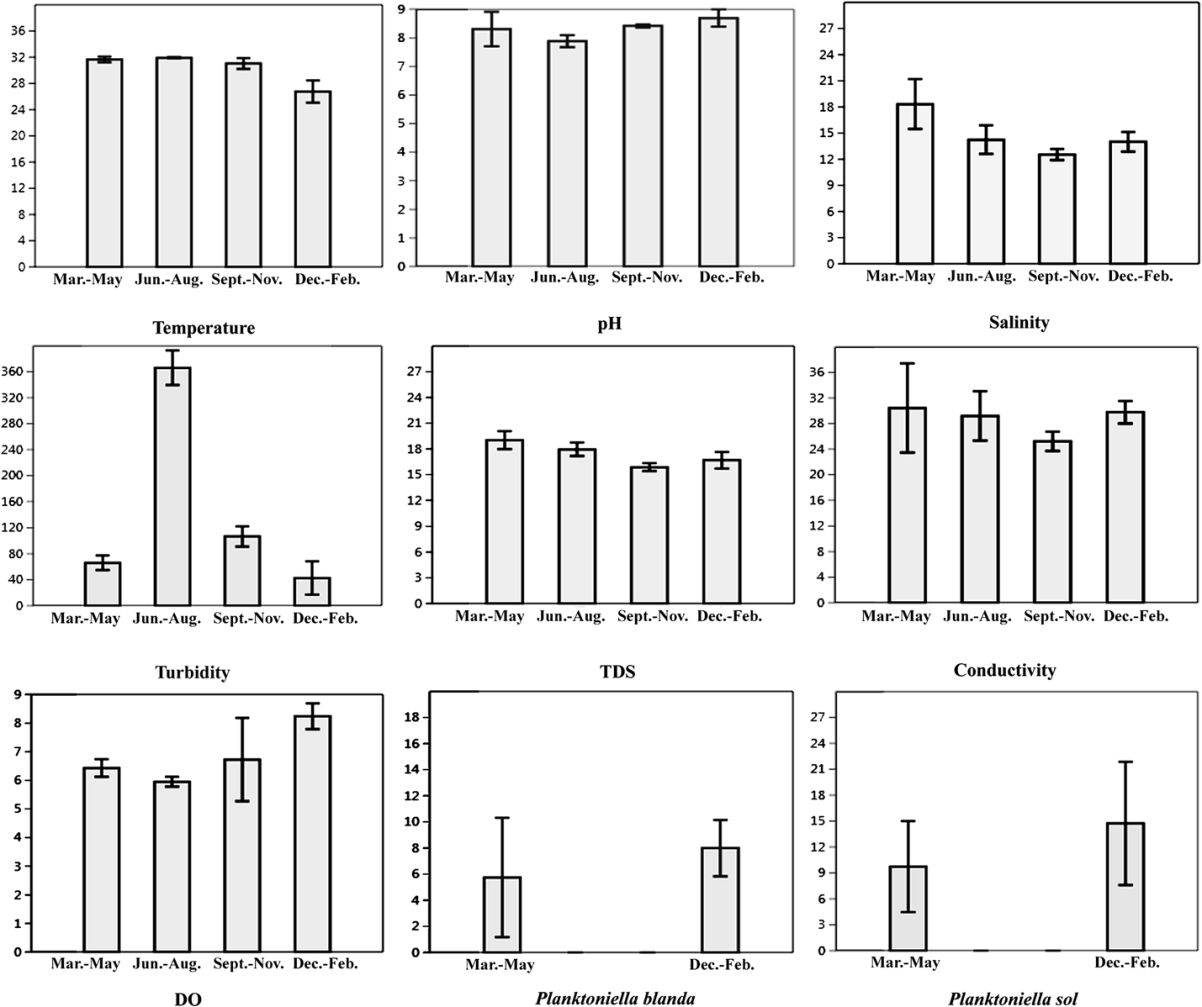
Bar Graphs of seasonal variation in mean and standard deviation of physico-chemical parameters and *Planktoniella* spp. cell count of the composite samples of the riverine stretch

The seasonal dynamics of the river stretch sampled was determined by consolidating each of the 4 sets of site-based data as single composite data for each parameter (Fig. 2). Noticeably higher standard deviation and variances in the seasonal dynamics noted in case of turbidity (145.6 ± 135.0NTU, *s*^2^ = 18215.4), conductivity (28.68 ± 4.27 mS/cm, *s*^2^ = 18.21), salinity (14.79 ± 2.73 ppt, *s*^2^ = 7.45) and also in the total annual counts of *P. blanda* (3.44 ± 4.29, *s*^2^ = 18.40) and *P. sol* (6.13 ± 7.68, *s*^2^ = 59.05).

**Fig. 3:**
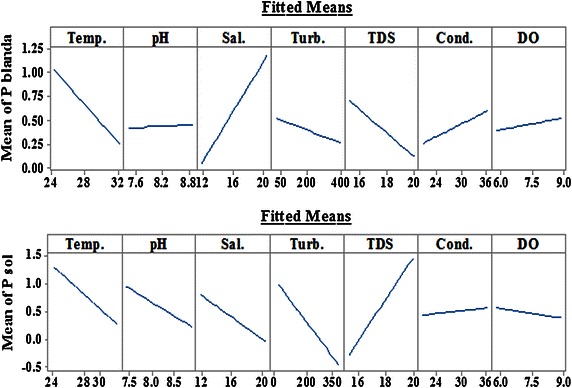
Main effect plots for *Planktoniella blanda* and *Planktoniella sol*

The absence of both *P. blanda* and *P. sol* during Jun.–Aug. and Sept.–Nov. in the samples examined is noteworthy, since it implies a very low population density. This is found to coincide with raised temperatures and turbidity and also with a steady decline in salinity of the surrounding environment (Fig. 2). Main effect plots based on Regression Model generated for *P. blanda* and *P. sol* show the extent to which presence and abundance of *P. blanda* and *P. sol* are governed by all the factors under consideration (Fig. 3). The plots for both *P. blanda* and *P. sol* indicate pronounced effect of even slight temperature variations on mean cell count (cells/ml) of the two species. Other than temperature *P. blanda* is indicated to be influenced by salinity, TDS, turbidity and conductivity. In case of *P. sol*, stronger influences are indicated in terms of TDS, turbidity, salinity, pH and conductivity.

Significance levels of Pearson correlation between physico-chemical parameters and *Planktoniella* spp. cell counts show highest significant negative correlation between *P. sol* versus temperature (at α = 0.01, p = 0.001) and *P. blanda* versus temperature (at α = 0.05, p = 0.037) (Table 2).

**Table 2:** Pearson correlation of physico-chemical parameters and *Planktoniella* spp. cell count

	Temp.	pH	Salinity	Turbidity	TDS	Cond.	DO	*P. blanda*
pH	−0.589*							
Salinity	0.214	−0.116						
Turbidity	0.490	−0.633**	−0.191					
TDS	0.336	−0.136	0.870**	0.160				
Cond.	−0.096	−0.183	0.190	0.033	0.275			
DO	−0.750**	0.488	−0.089	−0.548*	−0.240	0.023		
*P. blanda*	−0.524*	0.179	0.345	−0.579*	0.072	0.347	0.569*	
*P. sol*	−0.725**	0.373	0.298	−0.580*	0.078	0.289	0.533*	0.856**

Values in bold indicate the factors considered in the subsequent model

* Significant at 0.05 level; ** Significant at 0.01 level

The other factors that are found to have significant influence include *P. sol* versus turbidity (at α = 0.05, p = 0.019), *p. sol* versus DO (at α = 0.05, p = 0.034) and *P. sol* versus *P. blanda* (at α = 0.01, p = 0.000), showing significantly high positive correlation between the two species, *P. blanda* versus turbidity (at α = 0.05, p = 0.019) and *P. blanda* versus DO (at α = 0.05, p = 0.021). Though the Main Effects Plots indicate the influence of salinity on *P. blanda* and *P. sol*, it has not been included in the model since significant correlation was not found with Pearson correlation.

Analysis of Variance of multiple regression for both the species were generated with selected predictors with minimum p-values at α = 0.05 or 0.01 levels, before model selection. The summary for ANOVA of multiple regression for *P. blanda* and *P. sol* versus temperature, turbidity and DO give low values of S. ‘S’ is an estimate of the standard deviation of the error term in the model and is measured in the units of response variable. Lower values of S imply better prediction by the equation (Table 3). High values of R^2^ also obtained from summary of ANOVA of multiple regression. Value of R^2^ is significant in determining the ability of how the model fits the data, higher the value of R^2^ better is the way the model fits the data.

**Table 3:** Summary of ANOVA for *P. blanda* and *P. sol* with selected predictors

Name of species	DF	S	R^2^ (%)	R^2^ (adjusted) (%)
*P. blanda*	15	1.052	97.59	93.98
*P. sol*	15	2.191	96.20	91.87

The regression equation generated for *P. blanda* with *p* value = 0.000:

$$P. \, blanda = - 2.6 - 0.425 {\text{Temp}} . { } - 0.00082{\text{ Turb}} . { } + 0.255 {\text{DO}}$$

The regression equation generated for *P. sol* with p value = 0.000:

$$P. \, sol = 29.4 - 0.302 {\text{Temp}} . { } - 0.00161 {\text{Turb}} . { } - 0.682 {\text{DO}}$$

### Analysis of seasonal dynamics of biological factors

**Plate 2:**
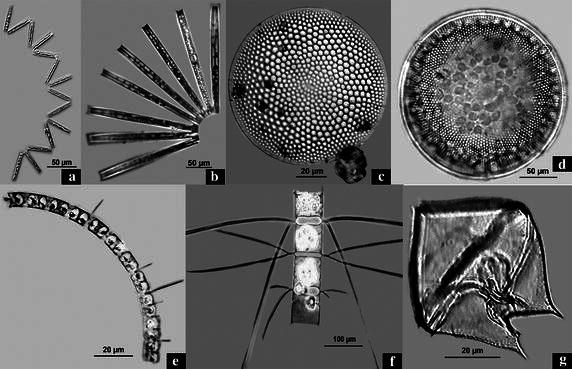
Light Micrographs of dominant phytoplankton forms at the sampling sites. **a**, **b** *Thalassionema nitzschioides* (Dominant 1); **c** *Coscinodiscus argus* (Dominant 2); **d** *Coscinodiscus centralis* (Dominant 3); **d** *Chaetoceros pseudocurvisetus* (Dominant 4); **f** *Chaetoceros lorenzianus* (Dominant 5); **g** *Protoperidinium pallidum* (Dominant 6)

The species of *P. blanda* and *P. sol* do not contribute heavily in numbers to the total phytoplankton community and in all the samples other organisms mostly belonging to the Bacillariophyceae or sometimes Dinophyta are found to be dominant (Table 4, Plate 2). The species that existed as more than 25,000 cells/l on an average are considered as dominants and have been taken into consideration in this particular work. The species that existed at levels lower than the above mentioned threshold have been avoided during this analysis.The average cell count/ml of the 6 dominant species during different seasons at the 4 sampling sites indicate an extremely high value of 100.6 cells/ml for *Protoperidinium pallidum*, a dinoflagellate during winter at site 1 (Table 4). This bloom like situation occurred only once during this entire study. The rest of the seasons and sites including site 1 in other seasons witnessed the abundance of *Chaetoceros pseudocurvisetus* Mangin, *Coscinodiscus argus* Ehrenberg, *Thalassionema nitzschioides* (Grunow) Mereschkowsky, *Coscinodiscus centralis* Ehrenberg and *Chaetoceros lorenzianus* Grunow.

**Table 4:** Seasonal dominance and average cell count of dominant phytoplankton in samples from the sampling sites

	Name of species	Months of dominance	Average cell count/ml
Dominant 1	*Thalassionema nitzschioides* (Grunow) Mereschkowsky	Mar.–May	52.0
Dominant 2	*Coscinodiscus argus* Ehrenberg	Jun.–Nov.	46.0; 65.0
Dominant 3	*Coscinodiscus centralis* Ehrenbergh	Jun.–Nov.	32.0; 29.6
Dominant 4	*Chaetoceros pseudocurvisetus* Mangin	Dec.–Apr.	37.6; 82.5
Dominant 5	*Chaetoceros lorenzianus* Grunow	Dec.–Apr.	28.9; 36.6
Dominant 6	*Protoperidinium* *pallidum* (Ostenfeld) Balech	Dec.–Feb.	100.6

The Jun.-Aug. and Sep.-Nov. periods harbour in plenty the two dominant species of *Coscinodiscus*—*C. argus* and *C. centralis*. Interestingly, these two seasons (monsoon and post monsoon) are also witness to absence of *P. blanda* as well as *P. sol* in the samples analysed, though presence cannot be ruled out under such circumstances.(Table 5). The dominance of *Thalassionema nitzschioides*, *Chaetoceros pseudo curvisetus* and *Chaetoceros lorenzianus* and even the bloom of *Protoperidinium pallidum* on the other hand do not have any detrimental effect on the two species of *Planktoniella*.

**Table 5:** Total cell count of *Planktoniella* spp. and dominant phytoplankton forms in 100 µl samples

Season and site		*P. blanda*	*P. sol*	Dominant 1	Dominant 2	Dominant 3	Dominant 4	Dominant 5	Dominant 6
Mar.–May	1	0	2	195	ND	ND	187	ND	ND
	2	7	13	217	ND	ND	0	ND	ND
	3	5	11	281	ND	ND	189	183	ND
	4	11	13	346	ND	ND	ND	183	ND
Jun.–Aug.	1	0	0	ND	145	ND	ND	ND	ND
	2	0	0	ND	187	ND	ND	ND	ND
	3	0	0	ND	190	130	ND	ND	ND
	4	0	0	ND	398	190	ND	ND	ND
Sep.–Nov.	1	0	0	ND	254	ND	ND	ND	ND
	2	0	0	ND	328	ND	ND	ND	ND
	3	0	0	ND	369	148	ND	ND	ND
	4	0	0	ND	366	ND	ND	ND	ND
Dec.–Feb.	1	8	13	ND	ND	ND	362	ND	503
	2	11	17	ND	ND	ND	412	109	ND
	3	7	6	ND	ND	ND	487	139	ND
	4	6	23	ND	ND	ND	390	186	ND

*ND* not found as dominant

Pearson Correlation of actual cell counts of the two species of *Planktoniella* with the six dominant forms yield significant relationships with *Coscinodiscus argus* (Dominant 2), *Chaetoceros pseudocurvisetus* (Dominant 4) and *Chaetoceros lorenzianus* (Dominant 5) along with a positively significant correlation between the two species of *Planktoniella* themselves (Table 6). Significant negative correlation exists in case of *C. argus* versus *P. blanda* (at α = 0.01, p = 0.001) and *C. argus* versus *P. sol* (at α = 0.01, p = 0.001). Significant positive correlation is found to exist in case of *C. pseudocurvisetus* versus *P. blanda* (at α = 0.05, p = 0.013), *C. pseudocurvisetus* versus *P. sol* (at α = 0.01, p = 0.007), *C. lorenzianus* versus *P. blanda* (at α = 0.01, p = 0.003) and *C. lorenzianus* versus *P. sol* (at α = 0.01, p = 0.002).

**Table 6:** Pearson correlation of cell counts of *Planktoniella* and the other dominant phytoplankton

	*P. blanda*	*P. sol*	Dominant 1	Dominant 2	Dominant 3	Dominant 4	Dominant 5
*P. sol*	0.856**						
Dominant 1	0.409	0.320					
Dominant 2	−0.751**	−0.747**	−0.507*				
Dominant 3	−0.391	−0.389	−0.264	0.587*			
Dominant 4	0.607*	0.646**	−0.127	−0.648**	−0.338		
Dominant 5	0.685**	0.712**	0.443	−0.596*	−0.311	0.547*	
Dominant 6	0.284	0.239	−0.144	−0.234	−0122	0.342	−0.170

* Significant at 0.05 level; ** Significant at 0.01 level

## Conclusion

*Planktoniella* spp. has been reported sporadically from the Indian Sundarbans, sometimes as part of the phytoplankton composition (Manna et al. 2010), sometimes as an element of decadal change (Biswas et al. 2010) and also in the context of extreme climate events like ‘*Aila*’ (Mukherjee et al. 2013, 2014). Nevertheless, this is the first morpho-taxonomical account of the genus *Planktoniella* from the Indian Sundarbans along with being the first exclusive analysis of its dynamics in the estuarine system of Sundarbans. The species being sensitive to temperature fluctuations, especially rise in temperature can be a favoured model organism in studying rising temperature effects. The sampling sites in the present study show an annual temperature range of 24.35 ± 6.43 °C (Dec.–Feb.) to 32.25 ± 1.48 °C (Mar.–May) both at site 4 and a maximum deviation of 6.43 also at site 4. This implies maximum temperature fluctuations in the southern most sampling site on the river stretch approaching the Bay of Bengal. This fluctuation also relates well with the presence/absence data of *Planktoniella* at different sites during different seasons. Negative correlation of high significance (at α = 0.01, p = 0.001) is noted in case of Pearson correlation of *P. sol* versus temperature and for *P. blanda* versus temperature the significance is at α = 0.05 and p = 0.037. Moreover, if the average deviation from mean of the seasonal temperature data is considered for each site, a gradual increase from north to south is noticeable: 1.91 (at site 1), 1.99 (at site 2), 2.36 (at site 3) and 2.75 (at site 4), implying that areas down south or nearer to the Bay of Bengal experience larger temperature fluctuations annually, compared to areas in the north or away from the Bay. Also noticeable is the fact that though the highest water temperature peak was noted during the period Mar.-May (32.25 ± 1.48 °C), which coincides with summer in the area, but a consistently high water temperature range was observed during Jun.-Aug., which coincided with monsoon in the area. Similar observations have been made by Biswas et al. (2010) who have reported that highest annual water temperature averages that were noted in the month of May during 2000, has shifted to the month of June in 2007. These indicate a gradual shifting of seasons with pronounced effect on organisms in the area.

Temperature is a much discussed factor known to influence the dynamics of phytoplankton populations and their distribution. It has also been discussed by many authors that phytoplankton cycles are basically controlled by water temperatures, but local weather and/or nutrient supply might have obscured this influence to some extent (McCombie 1953). Anderson (2010), Schindler et al. (1996), DeNicola (1996) have discussed in detail that temperature effects are most unlikely to be independent of other effects, particularly pH, nutrients, dissolved organic carbon, but the fact that each organism has its minimum, optimum and maximum limits of temperature tolerance remains undisputed. In this context, and considering the statistical implications in the present study temperature has been included in the models that speak about the dynamics of *P. blanda* as well as *P. sol*.

The samples from all the four sites were characteristically devoid of both *P. blanda* and *P. sol* during Jun.-Nov. which includes monsoon and post monsoon seasons in the area. This is noted with corresponding high temperatures and turbidity and also increased salinity. Similar observations with regards to taxa identified as *Planktoniella* spI and *Planktoniella* spII have been made by Manna et al. (2010) where no. of cells/ml of the two species increase gradually from November, 2008 to February, 2009 and with a sharp decline in March, 2009 become complete absent from the samples during April to May, 2009.

ANOVA of multiple regression with temperature, turbidity and DO as selected predictor variables yield regression equations for the two species of *Planktoniella* as response variables. The equation in case of *P. blanda* predicts that with each unit decrease in temperature, *P. blanda* count can increase by 0.425 considering the interaction of other predictor variables. Similarly, in case of *P. sol*, a single unit decrease in temperature can increase *P. sol* count by 0.302 in consistence with other predictor variables.

The organisms that are dominant in phytoplankton samples throughout the year in the studied area also exert their influence on the *Planktoniella* spp. Two species of *Coscinodiscus*—*C. argus* and *C. centralis* are found to be dominant during the period when both the species of *Planktoniella* are absent from the phytoplankton samples. On the other hand, bloom of *Protoperidinium pallidum* did not have any influence on the presence of *Planktoniella* and both the species were found to be present in their usual limited numbers in the samples. Likewise two species of *Chaetoceros*—*C. lorenzianus* and *C. pseudocurvisetus* dominant in phytoplankton samples could promote the presence of *Planktoniella* and exhibit strong positive correlation. Similar accounts of co-existence of *Planktoniella* with numerous other phytoplankton forms are found in literature.
